# Observational study of referrals to mental health from primary care

**DOI:** 10.1192/j.eurpsy.2025.1828

**Published:** 2025-08-26

**Authors:** A. Izquierdo De La Puente, R. Fernandez Fernandez, P. Del Sol Calderon, M. García Moreno

**Affiliations:** 1 Psiquiatria, Hospital Universitario Puerta de Hierro de Majadahonda; 2 Psiquiatria, Hospital Universitario Infanta Cristina, Madrid, Spain

## Abstract

**Introduction:**

Following the COVID pandemic, there has been an increase in mental health consultations in recent years, often referred to as a ‘fourth wave’. However, what has been observed in mental health centres is that these consultations are not so much about serious mental disorders, but rather occasional disorders due to social and work-related factors.

**Objectives:**

The aim of this study is to analyse the data collected from referrals to psychology in a mental health centre.

**Methods:**

Referrals to psychology from primary care at the Majadahonda Mental Health Centre were analysed for the period from October 2022 to April 2024 in a specific weekly consultation for the psychology waiting list.

The data collected are the diagnoses of these patients, whether or not they attend the assessment consultation and whether or not they are discharged after the first consultation.

**Results:**

A total of 115 patients were seen between October 2022 and April 2024. These patients were all notified of the appointment one week in advance.

Of the total number of patients who attended, 29 consulted for depressive-anxiety-adaptive disorder, 22 were referred for work-related reasons (burn out), 12 for marital problems, 7 for parenting problems, 12 had no clinical reason for referral to Mental Health and 25 did not attend the consultation.

Those who were discharged at the first consultation were 33.

**Conclusions:**

Given the scarce resources and long waiting lists, it is important at all levels of patient care not only to adjust expectations with regard to specialised care, but also to promote an appropriate setting. In this way it will be possible to run an efficient mental health care system.

**Disclosure of Interest:**

None Declared

